# Transforming community-based primary health care delivery through comprehensive performance measurement and reporting: examining the influence of context

**DOI:** 10.1186/s12875-024-02659-z

**Published:** 2024-12-04

**Authors:** Sabrina T. Wong, Manpreet Thandi, Ruth Martin-Misener, Sharon Johnston, William Hogg, Fred Burge

**Affiliations:** 1 School of Nursing, University of British Columbia, T201 2211 Wesbrook Mall, Vancouver, BC V6T 2B5 Canada; 2 Centre for Health Services and Policy Research, University of British Columbia, 201-2206 East Mall, Vancouver, BC V6T 1Z3 Canada; 3 School of Nursing, Dalhousie University, 5869 University Avenue, PO Box 15000, Halifax, NS B3H 4R2 Canada; 4 Department of Family Medicine, University of Ottawa, 201-699 Peter Morand Crescent, Ottawa, ON K1G 5Z3 Canada; 5 Lamont Primary Health Care Research Centre, Élisabeth Bruyère Research Institute, 43 Bruyère Street, Ottawa, ON K1N 5C8 Canada; 6 Montfort Hospital Research Institute, 713 Montral Road, Ottawa, ON K1K 0T2 Canada; 7 Department of Family Medicine, Dalhousie University, 5909 Veterans’ Memorial Lane, Abbie J Lane Building, Halifax, NS B3H 2E2 Canada

**Keywords:** Community based primary health care, Primary care, Learning health systems, Performance reporting

## Abstract

**Background:**

Community-based primary health care represents various community-based health care (CBPHC) models that incorporate health promotion and community development to deliver first-contact health services. Learning health systems (LHSs) are essential for improving CBPHC in which feedback from relevant stakeholders is used to continuously improve health systems with the goal of achieving population health and health equity. Performance reporting is one way to present data to clinicians and decision makers to facilitate a process of reflection, participation, and collaboration among partners to improve CBPHC.

**Methods:**

Our objective was to obtain feedback on a regional CBPHC performance portrait through key informant interviews. We used purposive convenience sampling to recruit participants who were clinicians in primary care and/or decision-makers in primary care at a regional level. The performance portrait summarized results of survey questions asked of patients, providers, and primary care organizations. The portrait was organized by the 10 pillars of the Patient’s Medical Home (PMH) model. Interview questions specifically asked about portrait content, formatting, interpretability, utility, and dissemination strategies. Content analysis was used to analyze interview data.

**Results:**

We completed 19 interviews with key informants from the Canadian provinces of Nova Scotia (*n* = 8), Ontario (*n* = 6) and British Columbia (*n* = 5). We coded transcripts into four content areas: (1) Usability as influenced by content and interpretability, (2) Formatting, (3) Utility, and (4) Dissemination. Using data and reporting back to clinicians and decision-makers about how their practices and jurisdictions are performing in primary care in meaningful ways is important. Our results suggest having available methodology notes, including the analysis used to develop any scoring, sampling and sample sizes, and interpretation of the statistics is necessary.

**Conclusions:**

This research was the first to create a comprehensive performance portrait using data driven by factors that are important to primary care partners. We obtained important feedback on the portrait in the context of usability, formatting, utility, and dissemination. This data needs to be used to provide feedback in continuous cycles to evaluate and improve CBPHC models as part of a LHS.

**Supplementary Information:**

The online version contains supplementary material available at 10.1186/s12875-024-02659-z.

## Background

Strong community-based primary health care (CBPHC) leads to a more equitable system of care with better population health outcomes at reduced cost [1, 2]. The Canadian Institutes of Health Research defines CBPHC as “the broad range of primary prevention (including public health) and primary care services within the community, including health promotion and disease prevention; the diagnosis, treatment, and management of chronic and episodic illness; rehabilitation support; and end of life care. CBPHC involves the coordination and provision of integrated care provided by a range of health providers, including nurses, social workers, pharmacists, dietitians, public health practitioners, physicians and others in a range of community settings including people’s homes, healthcare clinics, physicians’ offices, public health units, hospices, and workplaces. It is delivered in a way that is person- and population-centered and responsive to economic, social, language, cultural and gender differences” [3]. We use the term CBPHC to represent the various community based first-contact health care models that deliver general medical services as well as those incorporating health promotion and community development to address the social determinants of health.

CBPHC can be improved with the integration of learning health systems (LHSs), organizations where research and healthcare operations are blended to generate, synthesize, and refine evidence to improve population health, equity, patient experience, health workforce sustainability, and affordability [4]. With more and more data available in primary healthcare, there is also a need to build data presentation structures that can support LHSs, where decision makers, clinicians, and patients can use data in their improvement efforts. LHSs support learning from everyday care provision with the collection of high-quality clinical data [5] and feed the knowledge of what works best back to clinicians and other partners to create cycles of continuous improvement [6]. This feedback fuels LHSs for the purpose of helping to create a health care system that is continuously improving to support population health and health equity [4].

There are several purposes of performance information including research, accreditation, practice management, quality improvement, and public reporting [7]. Performance reporting on the extent to which CBPHC meets its objectives ought to be used for collaborative learning and accountability towards improving healthcare delivery for achieving better population health, equity, efficiency, and quality of patient care [7]. Healthcare performance measurement plays a significant role in guiding decision-making of healthcare system partners for quality of care and quality improvement [8]. Barbazza et al. [8] discuss the actionability of performance reporting, specifically if indicators are “fit for purpose” (i.e., serving an intended decision-making function), and “fit for use” (i.e., getting the right information to the right people). The use of data is also influenced by factors such as ease of interpretation of data and readability.

The audit and feedback strategy within practices is one way to improve performance in primary care [9], based on the assumption that clinicians are prompted to modify their practice when performance feedback shows inconsistencies with desirable outcomes [9]. Yet, CBPHC performance reporting remains challenging because of the dearth of concise and synthesized information [10].

Another strategy is to make available performance reporting at a higher aggregated level. Performance reporting can facilitate collaboration among partners as they set a common agenda [11]. Regional case studies of performance reporting can influence quality improvement agendas and improve performance [12, 13]. Indeed, past work shows that public performance reporting may improve health care performance [14–18] as it has the potential to improve the quality of care, increase accountability, facilitate public participation in health care [19, 20], impact societal and professional values and direct attention to issues not currently on the policy agenda [19–21].

There are examples of national public reporting of CBPHC performance in other countries such as Australia [22], United States of America (USA) [23], and the United Kingdom (UK) [24]. For example, the Australian government provides a performance dashboard related to the broad objectives of the National Healthcare Agreement [25]. Another example is the Bureau of Health Information that independently reports on the performance of the New South Wales healthcare system to inform improvements to care for patients, and enhance transparency and accountability [26]. However, there are limited efforts in Canada, most of which are provincial. The purpose of this work, known as TRANSFORMATION (http://www.transformationphc.ca/about/) was to improve the science of primary care performance reporting. One objective was to create a portrait of primary care service delivery to provide a high-level overview of CBPHC performance in three Canadian provinces: British Columbia (BC), Ontario (ON), and Nova Scotia (NS). This study informs which data is essential to report in CBPHC and how to best present it in order to support effective LHSs that align with regional governance structures in healthcare.

## Methods

TRANSFORMATION was a Canadian Institutes of Health Research funded team grant. It was a multi-provincial project seeking to influence policy through improving measurement and performance reporting in primary care [https://transformphc.sites.olt.ubc.ca/about/]. This component of the TRANSFORMATION program of research was a multiple, comparative, embedded case study [27]. The specific objective was to obtain feedback on a regional CBPHC performance portrait (Appendix A) in order to enhance meaningfulness and actionability of the information. The portrait was built from previous stages of the overall TRANSFORMATION project and contains a high-level overview of primary care performance in BC, ON, and NS using data collected in 2018. Information contained in the portrait was complied from a range of sources that included patient reported experiences, provider experiences and practice characteristics.

We obtained feedback on this performance portrait through key informant interviews. Our research questions were:

1. What features of a performance measurement system in CBPHC are important to key partners (clinicians, patients, policy makers)?
2. What are the preferences of key partners for how performance measurement results should be reported (content, format, mechanisms, audience)?

The cases were chosen to be from similar geographic regions, known as peer group A [28] from each of BC (Fraser East in Fraser Health), ON (Eastern Ontario), and NS (Central Zone). While these geographic peer group regions have since changed, the areas were originally chosen because they corresponded to administrative divisions with some decision-making authority over health resource allocation. We selected BC, ON, and NS for their varied approach to CBPHC reform [29, 30] and because our investigators had established relationships with clinicians and decision-makers in these three provinces.

### Recruitment

Interview participants were recommended by lead co-investigators in BC, ON, and NS. We used purposive convenience sampling to recruit participants who were decision-makers in primary care at a regional level. Potential participants were sent a brief introduction to the study via email, providing an opportunity to accept or decline being contacted by a study team member. Initial email invitations were sent by the principal decision-maker leads within each region.

## Procedures

Participants were sent a draft of the regional portrait (Appendix A) to review prior to their interview, after receipt of their electronically signed consent form. The portrait summarized results of survey questions asked of patients, providers, and primary care organizations and were organized by the 10 pillars of the Patient’s Medical Home (PMH) model. The College of Family Physicians of Canada (CFPC) defines a PMH as “a family practice defined by its patients as the place they feel most comfortable presenting and discussing their personal and family health and medical concerns” [31, p.2]. The 10 pillars of a PMH include: administration & funding; appropriate infrastructure; connected care; accessible care; community adaptiveness & social accountability; continuity of care; patient & family centred care; measurement, continuous quality improvement & research; and training, education & continuous professional development. The report compared the performance of each of the three provinces for each pillar of the PMH. All scores were converted to a scale of zero to 100, with higher scores indicating better performance. The portrait reported scores for each pillar for each province and the 95% confidence interval for each score. It also highlighted where differences across provinces within a pillar were statistically significant.

Semi-structured interviews with participants occurred online via zoom (https://zoom.us/) and lasted between 30 and 60 min. Interviews began with a brief introduction regarding the purpose, format, and confidentiality of the discussion. Interview questions (Appendix B) were designed to elicit information on portrait content, formatting, interpretability, utility, and dissemination strategies. Interview data were anonymized with a study identification number, and data access was restricted to only research team members.

Interviews were recorded and transcribed verbatim. Content analysis was used to analyze qualitative data from the interviews using NVivo software for data management. The coding structure was developed collaboratively by a research assistant and the leads from the three provinces. The interview audio files and transcripts were stored on the University of British Columbia’s workspace, a secure, on-campus cloud-based file sharing platform. Ethics approval was obtained in all 3 jurisdictions (University of British Columbia Behavioural Research Ethics Board: H18-02887; Ottawa Health Sciences Research Ethics Board: 20140458–01 H; Nova Scotia Health Research Ethics Board: 1017564).

## Analysis

Transcripts were coded into 4 content areas and relevant sub-content areas reflecting the interview guide. Table 1 provides a description of each content and sub-content area.

**Table 1:** Descriptions of content and sub-content areas

Content Area	Sub-Content Areas
**Usability as influenced by Content & Interpretability** Participants were asked questions related to the content of the CBPHC performance portrait.	**Interpretability** Participants were asked about the interpretability, specifically the ease of interpretability, of the portrait. **Recognizing Missing Elements and Drawbacks** Participants were asked about elements they thought were missing from the report, as well as some of the potential drawbacks influencing the use of the content. Participants also provided advice around improving usefulness of the portrait. Participants provided feedback on content included in the portrait that may not necessarily be useful.
**Formatting** *Participants were asked about the formatting of the portrait*, *generally*, *as well as whether anything could be done differently to increase usefulness.*	**Formatting Alternatives** (i.e. Hard Copy vs. Interactive) *Participants discussed the hardcopy format of the portrait in comparison to other formats such as interactive*, *websites*, *or infographics.*
**Utility** *Participants were asked how they might use the information presented in the portrait.*	**Usefulness and Benefits** *Perceived usefulness and benefits of the information presented in the portrait were discussed.* **Comparative Data** *Participants discussed how useful it is to compare data across regions of the provinces of British Columbia*, *Ontario and Nova Scotia.* **Optimal Frequency of Reporting** *Participants discussed how often they would like to see this type of report with updated data.* *Participants discussed how old data can be before it becomes less useful.*
**Dissemination** Participants were asked about audiences for this report. *Participants discussed their perceptions of who would benefit from receiving this report*, *as well as the optimal ways to deliver the report to them.*	

## Results

Research question 1 was answered in the discussion of content area 1 (usability as influenced by content and interpretability) where participants discussed which aspects of the portrait content was useful to them, as well as in sub-content area 1b (recognizing missing elements and drawbacks) where participants discussed whether there were any components missing from the portrait. Research question 2 was answered through discussions within all other content and sub-content areas.

A total of 19 of 20 (95% response rate) completed interviews in which we obtained their reflections on the CBPHC performance portrait. Eight participants were from Nova Scotia, five from British Columbia and six from Ontario. Table 2 shows the participants’ professional roles. All clinicians (*n* = 3) were family physicians. Decision makers (*n* = 16) operated at either regional or provincial levels and were involved with CBPHC performance monitoring and/or reporting.

**Table 2:** Participant regions and roles

Province	Clinicians’ Roles	Decision Makers’ Roles
**British Columbia (n = 5)** Clinicians: *n* = 1 Decision-makers: *n* = 4	Family physician in team-based care centre	• Management of learning, evaluation, and practice incentives • Senior nurse leaders • Leadership role within Ministry of Health • Leadership role within region
**Nova Scotia (n = 8)** Clinicians: *n* = 2 Decision-makers: *n* = 6	Family physicians	• Provincial role within local health authority • Provincial level responsibilities related to quality and practice support programs • Performance monitoring in particular zones and/or geographical regions • Recipient of CBPHC performance reports • Evaluator • Leader of quality improvement groups
**Ontario (n = 6)** Clinicians: *n* = 0 Decision-makers: *n* = 6	n/a	• Leadership position within Family Health Teams • Leadership role within Research and Evaluation department • Leadership role within Quality, Technology, and Performance department • Leadership role in Interprofessional Programs and Policy Department • Leadership role within CBPHC branch

Participants had leadership roles within either Ministries of Health or regional departments. At the Ministries of Health, participants were responsible for planning, developing, and implementing provincial strategies for primary care transformation, human resources, and funding models. Participants in regional departments filled a variety of roles that included research and evaluation; quality, technology and evaluation; interprofessional programs and policy; implementation of practice teams; and oversight of human resources within the primary care system.

Participants were in decision making roles that involved implementation of initiatives such as monitoring performance of collaborative family practice teams (NS03) or supporting the roll-out of collaborative family practice teams, patient medical homes, and/or primary care networks, sometimes including evaluation (NS01, BC01). Others were involved in the implementation and integration of primary care inter-professional team-based clinics, including nurse practitioner led primary care team-based clinics, and evaluating/reporting on their performance (BC02, ON01, ON02, ON04). Most participants stated they were examining and reviewing performance indicators to support resource allocation and equity in service distribution. These data ranged from examining patient reported experiences; panel sizes; indicators to administrative data such as visits and cancellations; and evaluations of quality improvement initiatives.

## Content area 1: usability as influenced by content and interpretability

The contents of the portrait were reported as clear, relevant, and useful in covering several areas of CBPHC. Participants found: (1) the organization of the content by PMH pillars provided a good foundational framework; (2) there was a good balance between being succinct and having enough detail to gain an understanding of CBPHC performance; (3) the content included relevant and important aspects of CBPHC. One participant stated,

“*I do like how it is organized by the elements of the patients’ medical [home model].*

*Certainly for the work that we do around the primary care delivery function of primary healthcare*, *that would be the kind of organizing framework that we would use as well…to kind of look at*, *as a system*, *how we’re performing under each of these elements” (NS01).*

Participants agreed that organizing the content using the PMH framework could help them move towards implementation of a more comprehensive model of primary care.

### Sub-content area 1a: interpretability

Most participants found the portrait straightforward to interpret. Participants agreed that explanations (e.g. score vs. a percentage, short paragraphs preceding graphs, bracketed statements under each question), colour-coding graphs, and breaking sections into more detail enhanced understanding of the portrait.

Most participants also emphasized some aspects of the portrait that were not as easy to interpret and provided suggestions to improve interpretability. One participant stated,

*“It’s a little bit hard to tell what goes into that composite score just from looking at it and one of the things that we struggle with in policy is making sure that we have the appropriate input in order to present data and findings” (ON05).*

This quote also points out challenges in reducing and simplifying complex information for stakeholders, including decision makers and their staff. Using scores and statistics need explanation as do concepts such as team-based care and comprehensive care. Having access to the items contributing to the score helped with interpretability.

## Sub-content area 1b: recognizing missing elements and drawbacks

We found a tension between providing high-level information in the form of a summary but that participants also wanted sufficient detail with regards to sampling, data collection and analytic methods and access to the survey questions.

One participant explained,

*“I think even on the first page a little text box…you could probably summarize it fairly succinctly with a couple of infographics to say the number of participants from each region*, *that date of data collection in each region*, *just a quick*, *little snapshot just to provide that context” (NS01).*

This quote also highlights the need to provide, in this case, the provincial contexts of primary care.

Generally, participants wouldn’t necessarily remove anything from the portrait. However, four participants thought that the statistics presented might not be useful for everyone. One participant thought the report may not be useful for people in rural areas, and two participants thought that questions about change may not be useful without more context. The following quote reflects this feedback:

*“I think if you have anybody that’s had any epidemiological training or even been sort of nudged in that direction*, *so most people*, *most nurses have taken a bit of a higher level of training and NP’s and GP’s would be able to appreciate that and anybody that’s into research*, *I’m not sure whether it has a lot of meaning for other people” (BC03*, *re: statistical data presented)*.

Developing a portrait tailored to clinicians or policy makers that balances the information and detail is a skill needed in conveying information.

## Content area 2: formatting

The majority of participants provided positive feedback on the formatting of the regional portrait. Participants’ comments included:

*“I love the way it was laid out. It was really easy to read and follow and I like the pillars that you picked. I like the way that it focuses on different dimensions and I think that the right ones are there… I actually don’t have anything to say about the format other than I like it” (NS06).*

*“It looks as though somebody thought a lot about not making it too difficult to read and paying attention to the fact that people have short attention spans and all those sorts of things. It probably achieves that and again*, *I recognize that we have many potential readers of this sort of information who probably have five minutes to scan over something and it’s trying to get something across in a very short period of time that is enough to maintain interest but still communicate something useful” (BC03).*

These comments also reflect on the right combination of explanatory text with graphs and that distilling a lot of information into small amounts of space while keeping it simple was helpful.

As one participant noted,

*“By the time I got to like the fifth page and if I’m imagining this as being four times bigger than it currently is with all ten areas being included*, *it would be really difficult to stay*, *like it’s kind of boring to see bar chart after bar chart and just text and bar chart and text and then bar chart*, *so I do know how to break that up… Obviously if you’re invested in the information that’s what you really want and when I kind of lock in to the content then I don’t care about the format as much as I’m invested in the content so maybe that’s okay for folks but it felt monotonous almost to read” (NS07).*

This quote reflects that both content and visual representation of data and text needs to keep reader attention. Providing key messages as an executive summary is important to convey content.

### Sub-content area 2a: formatting alternatives (i.e. Hard Copy vs. interactive)

The majority of participants preferred an interactive version of the information compared to a static or hard copy. An interactive version would allow for the ability to click on the indicators to get more detailed information about the data source, the specific survey questions, terminology definitions and how scores were calculated. Having the ability to compare regions across and within provinces was also seen as an advantage of an interactive version.

*“Interactive always works better because then people can go to wherever they want to learn. I think a static PDF document is not going to be helpful and I think in a situation like this we’ve got the 10 pillars and just*, *if you had the interactive click links and stuff like that*, *I also*, *because you’ve got bar graphs and things like that here*, *the more graphic rich you can make those I think the better for the reader” (ON01).*

This quote reflects the importance of being able to return to the information for further learning instead of a piece of paper being lost.

### Content area 3: utility

#### Sub-content area 3a: usefulness and benefits

Participants found the portraits useful in providing provincial comparisons and useful as an introductory document to dig deeper into content. One participant noted,

*“I think what you’ve done that I think is different from the other primary care measurement tools that I’ve seen is that you’ve translated these into a score…I think it’s definitely sort of a value added because I haven’t seen that*, *at least come across my desk in terms of another tool that does that kind of thing and then so having a score will allow you to understand your change over time more clearly.” (BC01)*.

Delivering data analytics within a framework familiar to many in primary care provided connection to participants’ current work. Participants thought that the content would help inform decision-making in some way, including re-emphasizing where attention needs to be focused. As one participant stated, beginning to report on performance in primary care is useful,

*“I know that we’ll use it. Where we’re at right now…obviously we report to the government and so our Deputy are very interested in understanding how we’re performing as a system” (NS05)*.

Another participant suggested,

*“…if it’s sort of an introduction document or an introduction study which then sort of leads to more in-depth stuff and maybe things that have actually helped to change things*, *then that’s useful” (BC03)*.

This quote also reflects components of the portrait that other participants commented on. Enhancing the utility of the portrait to provide additional resources to address challenges is helpful. For example, when presenting team-based care data, having resources for clinicians to draw on to improve team-based care would enhance the utility of the portrait.

### Sub-content area 3b: comparative data

Most participants found the comparative data useful. Participants wanted to understand similarities and differences across provinces. They saw it as an opportunity to learn from each other,

*“Those are some of the things that you want to see*, *like how did they improve sharing and*, *or how did they overcome certain barriers and things like that. Looking at what systems they are using? How are they using it*, *that*, *I think*, *is very helpful and it’s great to see this” (ON05)*.

The above quote highlights that decision makers and clinicians are wanting to understand common barriers and what others are doing to address PMH issues.

Some participants did not find cross provincial data useful. The quotes below highlight that clinicians have little time for reflection within daily activities of providing care or thinking about how to apply results in their clinic context,

*“My experience is that most family physicians day in and day out are challenged to get through their day of what work needs to be done without assessing these kinds of things within their practice” (NS08)*.

*“I don’t find that interprovincial comparisons are all that useful. I just have to be honest about that. I just find that it’s interesting to know about other provinces*, *but it’s not instructive… In terms of usefulness*, *I mean I would be more interested in local/regional type of comparisons within [province] than inter-provincial” (BC04)*.

### Sub-content area 3c: optimal frequency of reporting

Participants reflected on the burden of data collection versus seeing results. At the regional level participants suggested an annual report of patient experiences and outcomes was appropriate.

*“I guess from my operational hat I would say real time*, *right*, *in a perfect world*, *however… best case scenario is that this kind of thing would be refreshed annually*, *however then I know you get survey fatigue so even if it was every two years I think*, *would kind of be the minimum that we would look for” (NS01)*.

*“I think based on our own survey tools we don’t typically see any kind of change in less than a year. I think decision makers like to see data more frequently*, *but it just doesn’t change that often. I think at least annually would be great and probably more realistic than anything that’s more frequent” (BC01)*.

### Content area 4: dissemination

The information in the portrait would be most relevant for people working in primary care leadership positions at a regional and clinic levels, as stated by one participant,

*“Since this is provincial level data*, *I don’t know if sending it to clinicians directly would be very useful to them. I don’t think they’ll know what to do with it. If it was clinic level*, *yes but at a provincial level I would think anybody who is working more on the systems change side” (BC01)*.

The above quote highlights the need to achieve the right balance between sufficient granularity for clinic use by clinical leaders and aggregated data for provincial use by health system planners and decision-makers working in government or health authorities.

Some participants also highlighted working closely with primary care professional organizations so that they could use data in developing their training and resources.

*“I look at this*, *and I know this is more than just doctors*, *but I do look at this and I think of the Canadian College of Family Physicians*, *the patient medical home is their vision…It was their national college’s vision and I do think that they need to be charged with now at the local or provincial level at making it come to life and I think a lot of that will happen when they can see how they compare to their peers in other parts of the country. They’re absolutely the right audience” (ON01)*.

This quote above and the one below, suggest that dissemination of primary care portrait information through national and provincial organizations and patient partner organizations could be useful in moving primary care reform forward.

*“I think it would be interesting to get a patient perspective on it and I think you’d be probably surprised about how many patients are interested in terms of what is going on. I think that’s a good one. I think it’s more than just policy makers. I think there will be people that want to know what is going on. I think it’s going to be something that generally members of the public would be interested in as well actually” (NS04)*.

Dissemination of primary care information to clinics, regionally, provincially, and nationally is important in order to learn how one compares to other areas and understand where primary care reform is influencing patient reported experiences and outcomes of care.

## Discussion

A primary care portrait was produced using survey data collected from practices and their patients across three regions in BC, ON, and NS. Participants from all jurisdictions gave feedback on the usefulness of these data. Participants suggested that collecting and delivering the data in an organized framework such as the PMH model assisted with making the information relevant and a step towards a comprehensive model of primary health care. The PMH model reflects system integration goals and not just PHC goals and thus may be a useful foundational reporting structure for a LHS for each region. We discuss three main findings related to this work.

### Lack of consistent performance reporting

Canada has seen extensive reforms and investments in CBPHC totaling over $1 billion [32], unleashing a myriad of innovations, only some of which have been evaluated. While investment in primary care transformation has occurred there remains, in Canada, insufficient performance measurement and accountability and no national data infrastructure to regularly collect data [33]. In part, improving the organization and delivery of primary care continues, though largely uninformed by a variety of data sources [34]. The data needed to inform primary care remains largely inaccessible, tucked away in practices’ electronic medical records, walled off [34] or simply not collected regularly at a sufficiently granular level. At the time of data collection, this work was only the second largest attempt, after the 2014 Quality and Costs of Primary Care Survey [35], to collect patient reported experience and outcome measures within primary care practices in the past two decades. Indeed, the Canadian Institute for Health Information (CIHI) works closely to obtain regularly updated Commonwealth Fund (CMWF) survey data [36]. Results from the 2023 CMWF survey show that Canadians who have a regular primary care provider generally have more positive experiences (e.g. treated with courtesy and respect, spend enough time with them, etc.) when compared to the CMWF average across 10 high-income countries [37]. Importantly, collecting data regularly at the practice level is an important step to involve practices and decision makers at the level where quality of care can be improved [38] and in moving towards LHSs.

### Importance of performance reporting in driving quality improvement

Using data and reporting back to clinicians and decision-makers about how their practices and jurisdictions are performing in primary care in meaningful ways is important. Without collecting data or the analytics, organization and visual display of these data, deciding where to improve practice to better meet the need of patients remains challenging. A systematic review completed by Ivers, et al. [9] shows that audit and feedback on professional behaviour may be most effective if provided more than once, the feedback is provided by a supervisor or colleague verbally and in writing and includes clear targets and an action plan. Our work in developing these primary care portraits, organized using the PMH framework could be useful as practices and jurisdictions work to incorporate data into the quality improvement cycle. Our approach to primary care performance reporting created regional portraits that enable individual report provision to providers or regional aggregated reports. Ivers et al. [9] highlight that individual, rather than regional, reports are important for provider behaviour change. We did not test the PMH framework for the development of individual provider reports, however future work can address this.

### Balancing high-Level data and information science

Interpreting the results was enhanced with explanatory text of the graphs, definitions, and further detailed information for people to reflect upon. Using data to measure core constructs and enable clinic, regional and provincial comparisons could be useful. Reporting information to clinics, regional decision makers, professional organizations and patient partners was seen as important.

Provision of the “information science” along with the visual display of the data is important. Ensuring the data included in the portrait show key messages and align with partners’ performance/business objectives can reduce cognitive overload. Our results also suggest having available methodology notes is necessary, perhaps as an appendix, including: the items, analysis used to develop any scoring, sampling and sample sizes, and interpretation of the statistics. Additionally, use of primary care portraits depends on engaging end-users and building trusted partnerships between research and care delivery systems [39]. Our work provides critical steps to making visible primary care information at a practice and jurisdiction level. Clinicians, decision makers and patients (who answered our surveys) are vital partners in accelerating learning with the use of data.

### Limitations

This work has created knowledge and infrastructure to report on primary care performance. However, the work is limited in that there were fewer clinicians who participated in the interviews compared to decision makers. Future work should include more clinicians to ensure the design of primary care portraits meets their needs. Only participants from the three regions in BC, ON and NS participated which could limit the generalizability of the content in the portrait. Our sampling methodology could have restricted some partners from participation and overlooked other groups (e.g. patient groups, primary care networks) and thus, it is possible that information needs would vary.

### Future applications and actionability

In order to facilitate practical use and applicability of a performance portrait, it is necessary to consider several factors in addition to optimizing the report to meet key partner needs. For example, the allocation of time to spend reviewing data; incentives for partners to use the data; and prioritizing individuals who can be change champions and support clinicians and decision-makers are all key factors that need to be considered if the portrait were to be applied in practice and beneficial to quality improvement in primary health care. Further research is necessary to examine the real-world application of the performance portrait, perhaps by distributing it to key partners and engaging with them regarding acceptance, and facilitators and inhibitors to its use.

## Conclusion

This program of research is the first attempt to create a comprehensive performance portrait by using and linking data driven by what is most important to primary care partners. We have created a rich portrait of performance of CBPHC at a regional level and evidence of how well it is received by key stakeholders in primary care across the three provinces of BC, NS, and ON. In order to improve CBPHC, we need to integrate its evaluation within an LHS. Performance reporting within CBPHC has the potential to assess, improve, and evaluate quality of care, accountability, and ongoing participation in primary health care system reform. Our study obtained feedback on a regional CBPHC performance portrait from primary care clinicians and decision makers in the context of usability, formatting, utility, and dissemination. This valuable data needs to be used to provide feedback back to those working within primary care to create cycles of continuous improvement.

## Appendix A: regional performance portrait

[Please see file attached as a supplemental file]

## Appendix B: participant interview questions

**Table:** 

**Background questions** 1. Tell us about your role in [if decision-maker or provider] / experience with [if patient] primary healthcare? 2. [if decision-maker or provider] Do you or any of your team have performance reporting responsibilities? Can you tell me more about this?	
**Portrait questions** Here is a sample of a portrait that shows comparisons of your region with comparable health regions in other provinces. 1. What are your views about how the portrait could inform the decisions you or your organization has to make about primary care policy and or practice? 2. Content: a. From your perspective, is there anything that you want to know that is missing that could make the report more useful? b. Is there anything that is not useful to include? c. This report shows performance in your region compared to performance in other regions. What do you think about this? [point to an example of a regional comparison] How useful is it for you or organization to know this information? 3. Formatting: a. Is there anything that could/should be done differently in how the report is formatted or in the content of key messages or how key messages are created that would make the report more useful to you? b. What do you think of this format (hard copy of PDF), versus other formats (e.g. interactive websites, infographics?) 4. Interpretability: a. The report includes information about the context of primary care in each region that could affect performance. Let’s look at team-based care as an example. How would you interpret this information? Is it useful to include? 5. Utility: a. You have told me some important information about the usefulness of this performance data. Is there anything you want to add about potential benefits or drawbacks to the usefulness of the information in the portrait? b. How often would you want to see this type of report with updated data? c. How old can data be before it is less useful? d. If you had one piece of advice for us in developing the regional portrait so that it is more useful for you, what would it be? 6. Dissemination: a. What “audiences” [prompt: clinicians, government policy makers, health authority administrators, the public, etc] should receive the portrait and how [Prompt: available on a website; or social media sent via email; mailed as a hard copy]?	

## Electronic supplementary material

Below is the link to the electronic supplementary material.

Supplementary Material 1

## Data Availability

All data generated or analyzed during this study are included in this published article. Any further requested information is available from the corresponding author on reasonable request.

## Abbreviations

CBPHC: Community-Based Primary Health Care
LHS: Learning Health System
PMH: Patient’s Medical Home
USA: United States of America
UK: United Kingdom
BC: British Columbia
ON: Ontario
NS: Nova Scotia
CFPC: College of Family Physicians of Canada
CIHI: Canadian Institute for Health Information
CMWF: Commonwealth Fund
