# Supplementary material for: Transforming community-based primary health care delivery through comprehensive performance measurement and reporting: examining the influence of context
Source: BMC Prim Care. 2024 Dec 4;25:410. doi: 10.1186/s12875-024-02659-z (PMC11616284; doi:10.1186/s12875-024-02659-z)
Supplement: Supplementary file 1 — Supplementary Material 1 [file 12875_2024_2659_MOESM1_ESM.pdf]

DRAFT

TRANSFORMATION

Primary Health Care Performance Portrait

A stronger primary health care system is one that yields better health outcomes for Canadians at a lower cost. A key part of improving the primary health care system is to measure how it performs. This portrait provides information on primary care performance in three provinces, based on survey results from three regions: Fraser East (BC); Eastern Ontario (ON); and Central Zone (NS). Information is compiled from a range of sources and perspectives, including patients, providers, and primary care organizations, and for multiple dimensions of primary care performance. We compared the performance of the three provinces using the ten pillars of the Patient's Medical Home (PMH).

ADMINISTRATION & FUNDING	Practices need financial support delivered through appropriate remuneration models that enable governance, leadership, and management.	p. x
APPROPRIATE INFRASTRUCTURE	Physical space, staffing, electronic records and other digital supports, equipment, and virtual networks facilitate the delivery of timely, accessible, and comprehensive care.	p. 3
CONNECTED CARE	Practice integration with other care settings and services across the health care system, a process enabled by effectively integrating health information technology.	p. x
ACCESSIBLE CARE	Advanced and timely access, virtual access, and team-based approaches ensure care that's there when it's needed.	p. x
COMMUNITY ADAPTIVENESS & SOCIAL ACCOUNTABILITY	A PMH responds to the needs of a community it serves on the patient, practice, community, and policy level.	p. x
COMPREHENSIVE TEAM-BASED CARE	A broad range of services is offered by a well-connected interprofessional team. The team might not be co-located but the patient is always seen by a professional with relevant skills who can connect with a physician as necessary.	p. 6
CONTINUITY OF CARE	Patients live healthier, fuller lives when they receive care from a provider who knows them and how their health changes over time.	p. x
PATIENT- & FAMILY-PARTNERED CARE	Family practices respond to the unique needs of patients and their families within the context of their environment, involving them as active partners in care.	p. x
MEASUREMENT, CONT-INUOUS QI & RESEACH	Family practices strive for progress through performance measurement and CQI. Patient safety is always a focus, and new ideas are brought in through patient engagement.	p. x
TRAINING, EDU-CATION & CPD	Emphasis on training and education ensures that the unique knowledge and expertise of family physicians can be shared with the broader health care community, while ongoing development ensures constantly staying at the forefront of best practice.	p. x

TRANSFORMATION
www.transformationphc.ca @transformphc

Measuring and improving the performance of primary health care in Canada

DRAFT

Overview

Scores for each pillar of the Patient's Medical Home

Survey questions asked of patients, providers, and primary care organizations were mapped to the ten pillars. All scores were converted to a scale of zero to 100, with higher scores indicating better performance. Because the survey questions mapped to each pillar are quite different, it is more meaningful to compare the performance of provinces within a pillar rather than to compare across pillars. We report scores for each pillar for each province and the 95% confidence interval for each score. We also highlight where differences across provinces within a pillar are statisitcally significant.

Overall pillar scores, by province

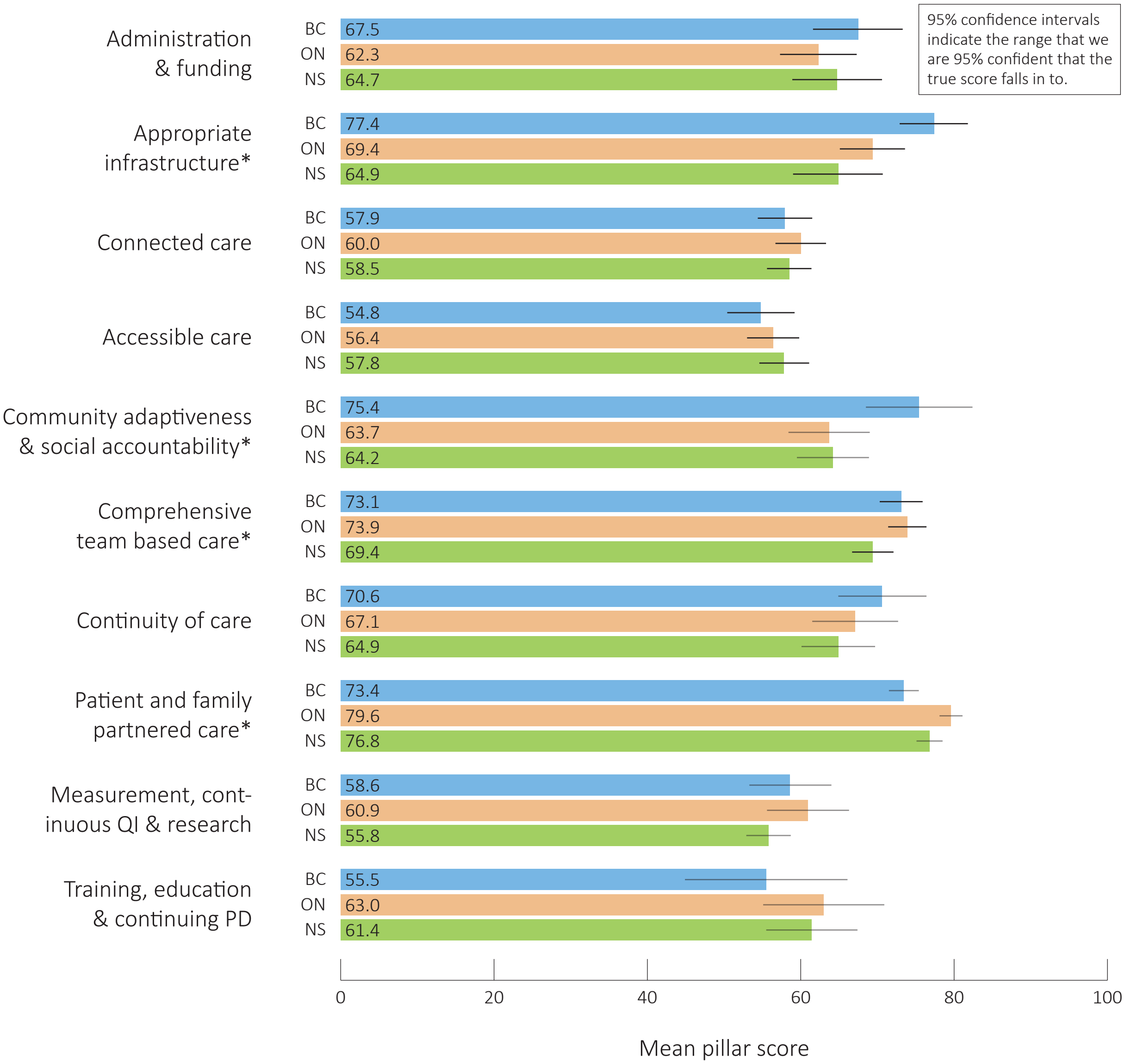

*Score is significantly different across provinces (p<0.05)
Horizontal lines indicate 95% confidence intervals

DRAFT

Appropriate infrastructure

Physical space, staffing, electronic records and other digital supports, equipment, and virtual networks facilitate the delivery of timely, accessible, and comprehensive care.

- Implementation of electronic medical records (EMR) is high in all three study regions. However, issues such as time, cost, interoperability, and privacy concerns challenge implementation.
- Case study participants described systems that allowed them to support patients or connect with external partners such as pharmacies, hospitals and labs.

Overall score

BC has the highest overall score for this pillar:

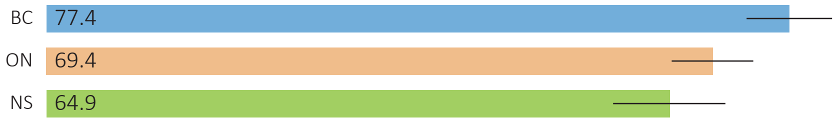

Detail

To examine this pillar, we examined the data from clinics and providers. Clinics reported on the characteristics of their practice, and of the services and procedures they provided to patients. Clinicians completed surveys to describe their experience of working at their clinic both as an individual and as a member of an interprofessional team.

Clinic-reported data

Access to computer software and tools | Whether or not providers working at the practice had access to computer software which could aid in a variety of tasks, such as medical decision-making or interfacing with diagnostic imaging laboratory services. This could also include software that supports scheduling or conducting appointments with patients.
[A higher score indicates more access]

The numbers provided are estimates and standard deviations for the estimates, indicating how much responses varied.

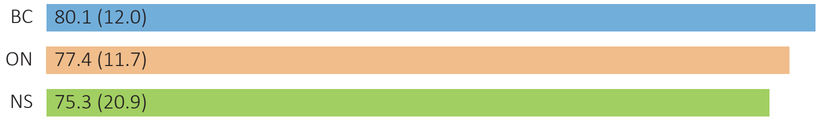

Medical record system | How easy it was to use the current patient medical record system to generate useful outputs including lists of patients organized by specific criteria such as diagnosis, or clinical summaries that providers could give to patients.*
[A higher score indicates greater ease]

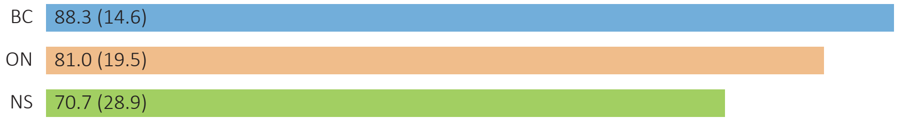

*Score is significantly different across provinces (p<0.05)
Horizontal lines indicate 95% confidence intervals

DRAFT

Computerized medical record system | Whether or not the medical record system was computerized
[A higher score indicates greater computerization]

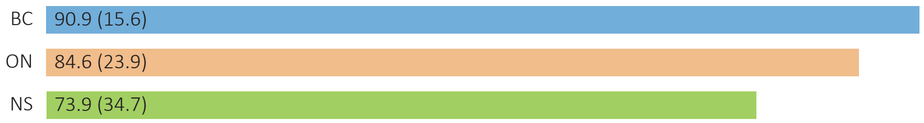

Reminder systems | Whether the clinic had a system to provide regular reminders to patients and providers. These systems could prompt on tasks like reminders for regular screening tests, provide a checklist for preventative clinical practices, or help to change behaviours.*
[A higher score indicates the presence of a computerized system]

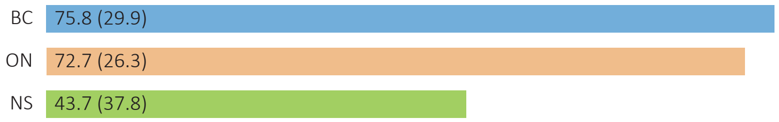

Change in information technologies access | The extent accessibility of information technologies was perceived as having changed over the past five years.
[A higher score indicates larger change]

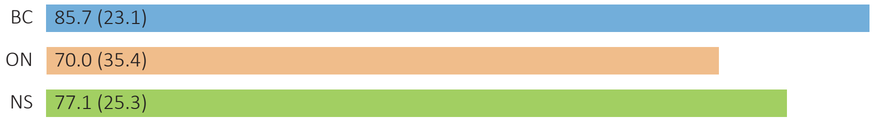

Change in availability of nurses | The extent to which the possibility of having nurses on the team had changed over the past five years.
[A higher score indicates greater availability]

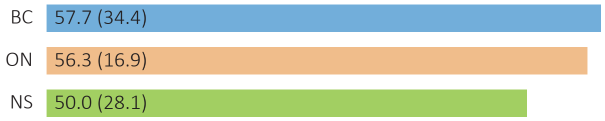

Possibility of physician recruitment | The extent to which the possibility of recruiting new physicians to the team had changed over the past five years.
[A higher score indicates greater possibility]

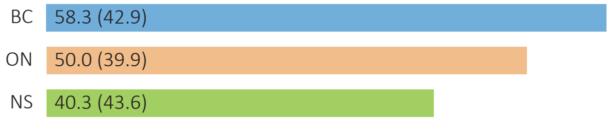

Sharing | Whether or not family physicians at the clinic shared resources such as staff, physical space, and medical record systems, as well as whether they shared operating costs.
[A higher score indicates greater sharing]

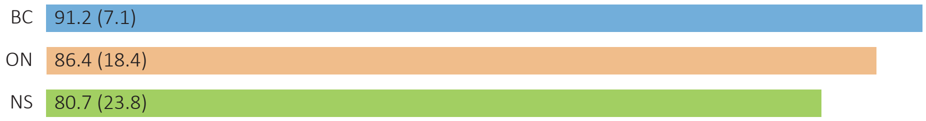

*Score is significantly different across provinces (p<0.05)

DRAFT

Online options for patients | Whether or not the clinic had online resources patients could access to request appointments, referrals, or prescriptions.*
[A higher score indicates a system with more options for patients]

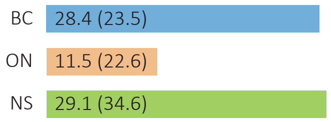

Provider-reported data

Electronic medical record (EMR) users | The frequency participants used their organization's EMR for tasks including ordering tests and viewing results, prescribing medications, or retrieving hospital records.
[A higher score indicates more users]

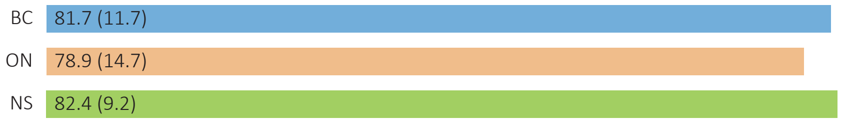

EMR documentation ease | The ease with which participants felt they could use their organization's EMR to document information about patients and patient visits.
[A higher score indicates a greater ease]

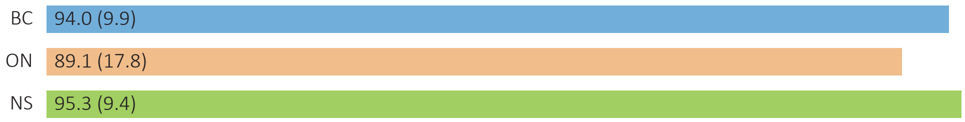

EMR search ease | The ease with which participants felt they could use their EMR to search for and find patient information.
[A higher score indicates a greater ease]

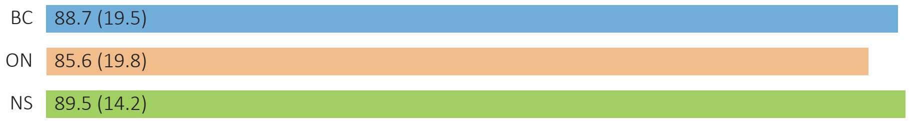

*Score is significantly different across provinces ($p<0.05$)

DRAFT

Comprehensive team-based care

A broad range of services is offered by a well-connected interprofessional team. The team might not be co-located but the patient is always seen by a professional with relevant skills who can connect with a physician as necessary.

- ON, BC, and NS have all introduced interprofessional team-based approaches. Implementation of interprofessional teams in ON is more formalized and supported by greater resources than in BC and NS. This includes: government funding, defined mandates and accountabilities, and formal team structure and governance requirements.
- In BC and NS, the most activity around team-based care is in rural areas. These jurisdictions are increasing the number of interprofessional team-based models.

Overall score

Ontario has the highest overall score for this pillar:

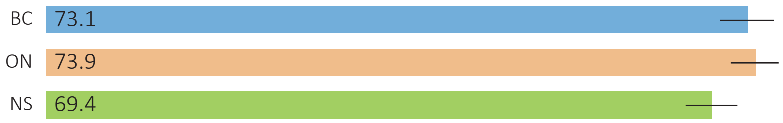

Detail

To examine this pillar, we examined the data from patients, clinics, providers and teams. Patients completed surveys to describe their experience and perception of interactions with their primary care team, and of the care they received from this team. Clinics reported on the characteristics of their practice, and of the services and procedures they provided to patients. Clinicians and teams completed surveys to describe their experience of working at their clinic both as an individual and as a member of an interprofessional team.

Patient-reported data

Health promotion discussions | Patients were asked whether a primary care provider discussed a variety of topics related to the social determinants of health and health behaviour with them.*
[A higher score indicates more frequent discussion]

The numbers provided are estimates and standard deviations for the estimates, indicating how much responses varied.

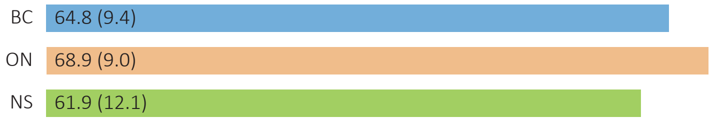

Managing health concerns | Patients were asked whether they felt the primary care team provided everything they needed to manage their own health concerns.*
[A higher score indicates more frequent provision of supports]

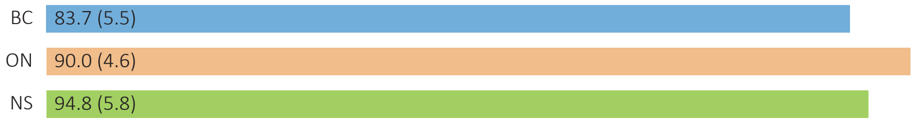

*Score is significantly different across provinces (p<0.05)
Horizontal lines indicate 95% confidence intervals

DRAFT

Team role clarity | Patients were asked about how they perceived interactions between members of their primary care team. This included whether the patient received conflicting messages from multiple providers, services provided by the team seemed coordinated, and the team seemed to work well together.*
[A higher score indicates less confusion and greater clarity among team members]

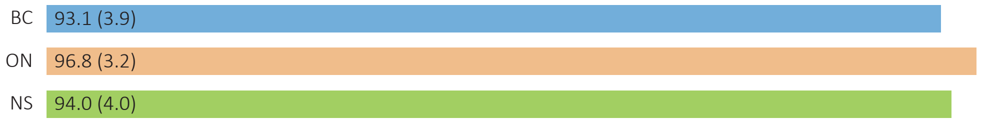

Clinic-reported data

Availability of routine procedures | Whether specific services were available on-site. These included strep test, skin biopsy, IUD insertion, and others.
[A higher score indicates more availability]

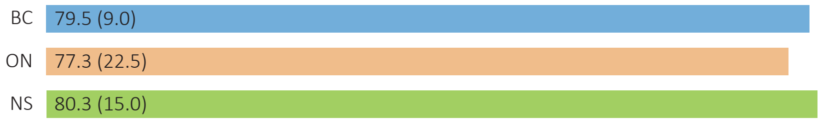

Availability of a range of services | Whether family physicians in the clinic provided a variety of general services, including: non-urgent routine care, home visits, and end-of-life care.*
[A higher score indicates a wider range of services]

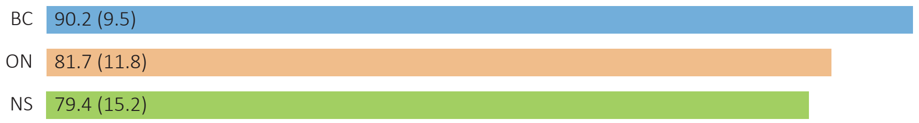

Role of registered nursing staff | A measure of the roles and functions carried out by registered nursing staff on the team.
[A higher score indicates a wider range of roles]

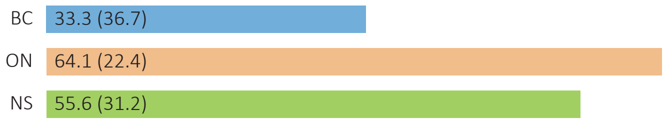

Frequency of meetings
[A higher score indicates meetings are held in the clinic more frequently to discuss business, clinical, or office operations issues]

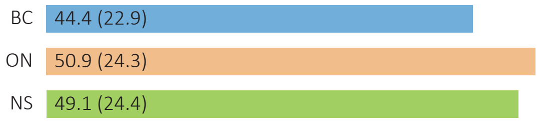

Communications to coordinate care | General descriptions of how care is coordinated among the professionals working at their site. This included questions about frequency of informal or ad-hoc exchanges between professionals at the clinic, pre-established protocols, case discussion meetings, or communication through EMRs.
[A higher score indicates more frequent communications across these four types]

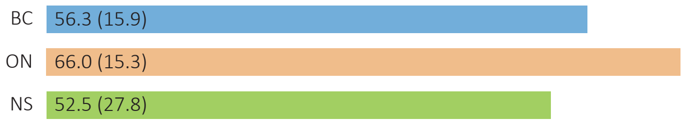

*Score is significantly different across provinces ($p<0.05$)

DRAFT

Change in teamwork | Whether the respondent felt teamwork among professionals at the site had changed during the past 5 years.
[A higher score indicates improvement]

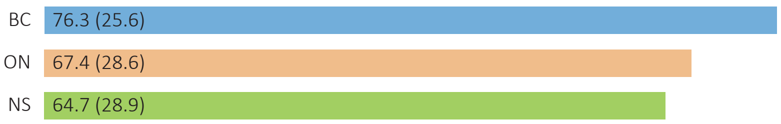

Provider-reported data

Scope of skills used | The extent to which participants felt they were working to full scope of practice.
[A higher score indicates having used a larger share of scope of practice]

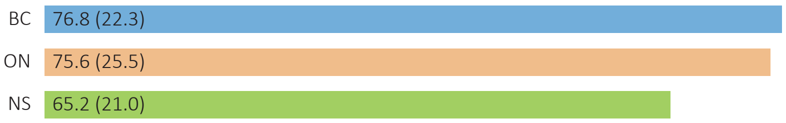

Efficiency of skills used | Whether they felt their skills were being used effectively.*
[A higher score indicates a more efficient use of skills]

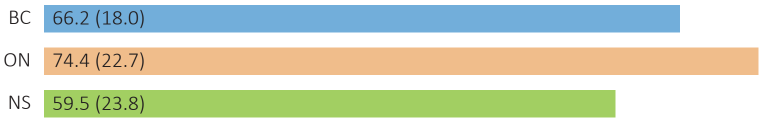

Value of colleague input | Whether they felt colleagues provided input that helped them work to the best of their ability.
[A higher score indicates more valued input]

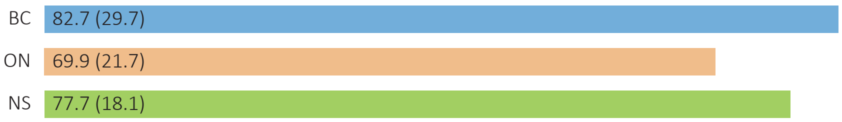

Team-reported data

Team participation | Team members were asked about their experience of the general climate among team members. This included questions about whether members kept in touch with each other, or whether they felt understood and accepted by colleagues.
[A higher score indicates more team participation]

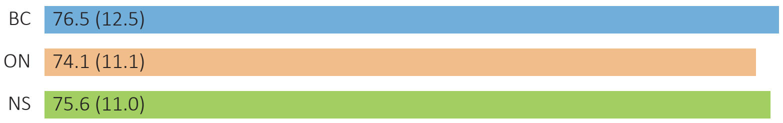

Team support for innovation | Team members were asked about their perception of the team's approach to innovation. This included questions about how the team approached new problems and whether or not they worked together to solve these problems.
[A higher score indicates more support for innovation]

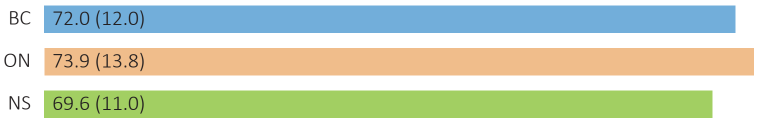

*Score is significantly different across provinces ($p<0.05$)

DRAFT

Clarity of team objectives | Team members were asked about whether their team had clear objectives, and the extent to which team members (themselves included) share a commitment to these objectives.
[A higher score indicates more clarity]

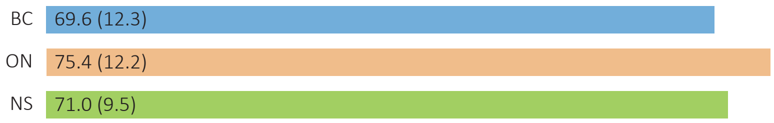

Critical reflection among team members | Team members were asked about whether their team shared ideas and/or engaged in critical reflection. For example, participants were asked whether the team shared ideas, questioned their overall direction, and discussed potential weaknesses in how they were approaching their work.
[A higher score indicates more sharing and reflection]

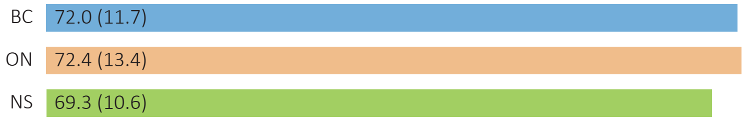

*Score is significantly different across provinces ($p<0.05$)
